# Electroncephalography in an ICU: indications and results

**DOI:** 10.1186/cc12650

**Published:** 2013-06-19

**Authors:** SP Cavalcanti, G Castro, L Pineiro e Pinho, RCC Lobo Ferreira, V de Carvalho Bacelar, R Silva de Carvalho, SM Pereira de Castro, P da Silva Sousa Carvalho

**Affiliations:** 1 Ceuma University, Renascença II, São Luís, MA, Brazil

## Introduction

Evaluation electroencephalography (EEG) in the ICU provides a noninvasive measure at the bedside of brain function in critically ill patients. The objectives of this study were to determine the main indications for electroencephalographic monitoring in ICUs, the possible findings, and clinical outcome of patients.

## Methods

This was a retrospective study analyzing the records of patients who underwent EEG study from October 2011 to October 2012. Data collection was performed with the aid of a standardized form, prepared by the authors, which consisted of the following variables: age, sex, indications for performing EEG, ICU admission diagnosis, electroencephalographic findings, and clinical outcome of the patient. After collection, the data were tabulated using Excel 2007. Later they were exposed in descriptive tables and the statistical analysis was performed using the Statistical Program 7.0, considering a significance level of 0.05.

## Results

We analyzed 35 EEGs in 29 patients; five underwent more than one examination. Of these patients, 17 (58.6%) were male and 12 (41.4%) were female. The mean age was 64.59 ± 17.91. With regard to clinical outcome, we observed that 79.3% of those assessed were epileptiform discharges and only 20.7% died. We found that acute neuronal injury was the most observed clinical diagnosis, accounting for 28.5% (AVEH, 17.1% and AVEI, 11.4%), followed by TBI (17.1%), severe sepsis (17.1%), toxic-metabolic encephalopathy (11.4%), status epilepticus (11.4%) and others (14.3%). No statistical significance occurred when we correlated diagnosis and mortality. The EEG was performed if the patient had an altered level of consciousness (62.7%), seizures (25.7%) or a combination of altered consciousness and convulsion (8.6%). Coma was present in 11.4% and only 2.9% had EEG evaluation for post PCR. The presence of epileptiform discharges was identified in 11 (31.4%), although most did not show this pattern (24, 68.6%). Of the 35 reports, seven (20%) had electrographic status and among these four (11.4%) had an epileptiform pattern while three (8.57%) met periodic pattern criteria. All patients had electrographic status resolution of seizures and were discharged.

## Conclusion

EEG shows great applicability in ICUs, guiding sound decisions in patients with impaired consciousness.

